# Quantitative analysis of commercial coating penetration into *Fagus crenata* wood using X-ray microtomography

**DOI:** 10.1038/s41598-024-64892-x

**Published:** 2024-06-17

**Authors:** Tyana Solichah Ekaputri, Takashi Tanaka

**Affiliations:** 1 The United Graduate School of Agricultural Science – Gifu University, 1-1 Yanagido, Gifu-shi, Gifu 501-1193 Japan; 2 College of Agriculture, Academic Institute, Shizuoka University, Ohya 836, Suruga-ku, Shizuoka, 422-8529 Japan

**Keywords:** Composites, Plant sciences

## Abstract

Recent advances in wood treatment include the use of eco-friendly coatings to improve the wood’s dimensional stability and appearance. Assessing coating performance during its service life is critical for establishing a knowledge base for product optimization. Numerous approaches, including microimaging, are available for analyzing coating behavior. In addition to conventional microscopic techniques, high-resolution X-ray microtomography is a tool that provides nondestructive imaging of coatings and their substrates. In this study, we performed two-dimensional (2D) and three-dimensional (3D) visualization of tomographic reconstruction images of two coating types, spray and brush, to observe and assess the distribution of several commercial Japanese coating materials in *Fagus crenata*. X-ray images and plot profiles were used to determine the penetration depths and thicknesses of coatings. Each coated sample was scanned using X-ray microtomography, which allowed successful visualization and quantification of the coating penetration depth. Chemical content and concentration of the coating materials influenced penetration depth and amount.

## Introduction

In recent times, thin covering layers have become an ubiquitous practice across scientific and industrial sectors. A thin layer applied to an object or substrate is referred to as a coating. Synergistic interactions between the substrate and the covering material occurred when coatings were applied^1^. Coatings can enhance physical, chemical, and aesthetic properties and lower the cost of the final wood product. Coatings such as varnish, stain, and paint are commonly used in the furniture industry.

Interactions between coatings and natural materials such as wood, along with the complex formulations of coatings, make predicting their service life and performance more challenging^2^. Coatings are used to achieve a synergistic effect between the substrate and covering materials^3^. It is well known that coating performance, particularly adhesion, depends on the hygroscopic characteristics of wood composite panels^4^. Therefore, the type of coating plays an important role in its performance, such as the film thickness, which affects abrasion resistance and adhesion. The finishing characteristics of coatings on wood surfaces depend on many variables, with the coating film thickness being a significant one^5^. Additionally, the penetration of the coating into wood is important for several reasons. First, coating penetration can improve water repellency and dimensional stability, and reduce surface cracks. Second, the adhesion of the coating to wood benefits from a certain degree of penetration, such as mechanical entanglement or interlocking, adsorption interactions by polar and dispersive forces, and intermolecular diffusion. Third, coatings containing fungicides may have improved the penetration of the fungicide because it penetrates together with the coating^6^.

A fundamental understanding of the interactions between wood and coatings is desirable. Regarding the wood surface, Salin^7^ stated that practically all wood species have a damaged surface layer, which causes variations in the layer's qualities. These variations alter the behavior of the piece, particularly when it interacts with the surrounding air or other media. The condition of a wooden surface is important for coating applications. In a previous study, apart from the anatomical structure of wood, coating penetration was mainly determined by the ability of the coating to flow into the lumina of either the tracheid or ray-cells^6^. The flow of a coating through wood capillaries is likely influenced by many variables, such as viscosity, surface tension, drying rate of the coating, and diameter of the wood capillaries. Thus, anatomical microscopic penetration has recently become well known.

X-rays are a non-destructive method that provides 3D images of internal structures and have been increasingly applied to wood. A study conducted by Ekaputri et al.^8^ demonstrated the applicability of X-ray microtomography to visualize the coating penetration phenomena in wood using two different X-ray targets: Cu and Mo. They successfully visualized the penetration of commercial Japanese coatings into Japanese beech wood. Additionally, Tanaka and Kawai^9^ described the potential of X-ray measurement for quantitative analysis by observing the dissolved CsCl concentration in wood during the drying process. Bulcke et al.^10^ proposed a detailed analysis of coating on and in the wood, suggesting that layer thickness and penetration depth can be calculated using X-rays as a non-destructive technique by emphasizing the essential step of image analysis: segmenting bilateral filtered scans using a multimodal normal distribution. With accurate segmentation, calculating the thickness and penetration depth of solvent and aqueous coatings on wood, such as Scots pine, which has a lower density than the coating itself, becomes straightforward. Bessieres et al.^11^ used X-ray tomography to determine the penetration of solvent-borne, waterborne, and powder coatings into various wooden substrates using grayscale evolution with the plot profile tool in ImageJ. They successfully implemented this method to characterize liquid solvents and water-based systems in wood. However, to the best of our knowledge, X-ray microtomography has not yet been employed to investigate the coating penetration phenomenon and provide the 3D quantification of wood. Therefore, this study aimed to determine the range and average penetration depth of selected commercial coatings used in Japan, using X-ray microtomography. 3D quantification of the coatings was performed. The applicability of X-ray microtomography for distinguishing between the anatomical elements of wood and coating materials using commercial Japanese coatings was evaluated. The effects of the coating type, application method, and chemical content are also discussed.

## Methods

### Sample preparation and coating application

Four rotary veneers of *Fagus crenata* Blume with dimensions of 300 mm × 300 mm × 1.0–1.3 mm were pre-treated in the constant humidity at 20 °C and 65% RH. The veneers were dried in a constant temperature oven (DKN812) for 24 h at 60 °C. After drying, the veneers were cooled for several minutes in a plastic bag to prevent changes in moisture content. They were then cold-pressed and hot-pressed at 125 °C with 1 MPa pressure for 20 and 4 min. The veneers were cooled in plastic bags and placed in a room with constant humidity and temperature for 24 h. After cooling, the veneers were cut into 12 veneers with dimensions of 100 mm^2^ using a table saw. One veneer was used as the control (no coating applied), six were coated using a commercial Japanese coating with a spray application, and two were coated using a brush application. Coating materials were purchased from Shizuoka, Japan. The specifications of the coating are listed in Table 1.

**Table 1:** Coating specifications.

Material Name	Color	Application	Coating base	Ingredients That Might Affect X-ray Interaction	Percentage (%)	Chemical Formula
Aspen Lacquer Spray	Brown (BR)	Spray	Oil-based	Titanium Dioxide	0.1–1	TiO_2_
	Gold (GL)			Brass (Copper/Zinc)	1.0–5.0	Cu/Zn
	Matte White (MW)			Amorphous Silica	1.0–5.0	SiO_2_
				Titanium Dioxide	1.0–10	TiO_2_
	Pink (PK)			Titanium Dioxide	1.0–10	TiO_2_
	Silver (SL)			Aluminum	1.0–5.0	Al
	White (WH)			Titanium Dioxide	1.0–10	TiO_2_
Outdoor xylem protective paint WOOD oil-based	Light Oak (LOO)	Brush	Oil-based	Amorphous Silica	0.1–1	SiO_2_
				Ferric Hydrated Iron Oxide	1.0–5.0	Fe(OH)O
				Cobalt 2-Ethylhexanoate	0.1–1	[CH_3_(CH_2_)_3_CH(C_2_H_5_)CO_2_]_2_Co
Outdoor xylem protective paint WOOD water-based	Light Oak (LOW)	Brush	Water-based	Amorphous Silica	0.1–1	SiO_2_
				Ferric Hydrated Iron Oxide	1.0–5.0	Fe(OH)O
				Titanium Dioxide	0.1–1	TiO_2_

Each coating material was applied to the face of the veneer (no lathe check caused by the blade during rotary cutting) with a 2-coat application at a spreading rate of approximately 280 g/m^2^, as suggested by Kinoshita^12^ as the typical spreading rate for topcoats on hardwood surfaces. For spray-type coatings, two applications were applied to the veneer at a distance of approximately 15 cm. The application interval for each spray coating was 30 min. In the case of brush-type applications, the interval before the second application was 1–2 days. These applications were applied to the samples according to the technical datasheets published by the manufacturers of the coatings. After coating, all veneers were air-dried for 24 h.

### Coating thickness measurement

A micrometer screw gauge was used for dry coating thickness measurements. The initial and dried coated veneer thicknesses were measured to calculate the coating thickness (dry conditions). The coating thickness was calculated using Eq. 1 and the results are listed in Table 2.

1 $$Dry \;Coating\; Thickness\; \left( {{\mu m}} \right) = Wood \;coated\; \left( {dry} \right)\; thickness\;\left( {{\mu m}} \right) - Wood\; initial\; thickness\;\left( {{\mu m}} \right)$$

**Table 2:** Coating thickness.

No	Sample color	Wood initial thickness (µm)	1st coat	2nd coat	Coating thickness (µm)
			Coated wood (dry) thickness (µm)	Coated wood (dry) thickness (µm)	
1	Brown (BR)	1002	1007	1019	17
2	Matte white (MW)	995	1018	1032	37
3	Pink (PK)	1019	1022	1034	15
4	White (WH)	985	995	1020	35
5	Gold (GL)	998	1009	1033	35
6	Silver (SL)	1028	1037	1054	26
7	Light Oak oil-based (LOO)	980	997	1011	31
8	Light Oak water-based (LOW)	958	987	1001	43

### X-ray microtomography scanning 

**Figure 1:**
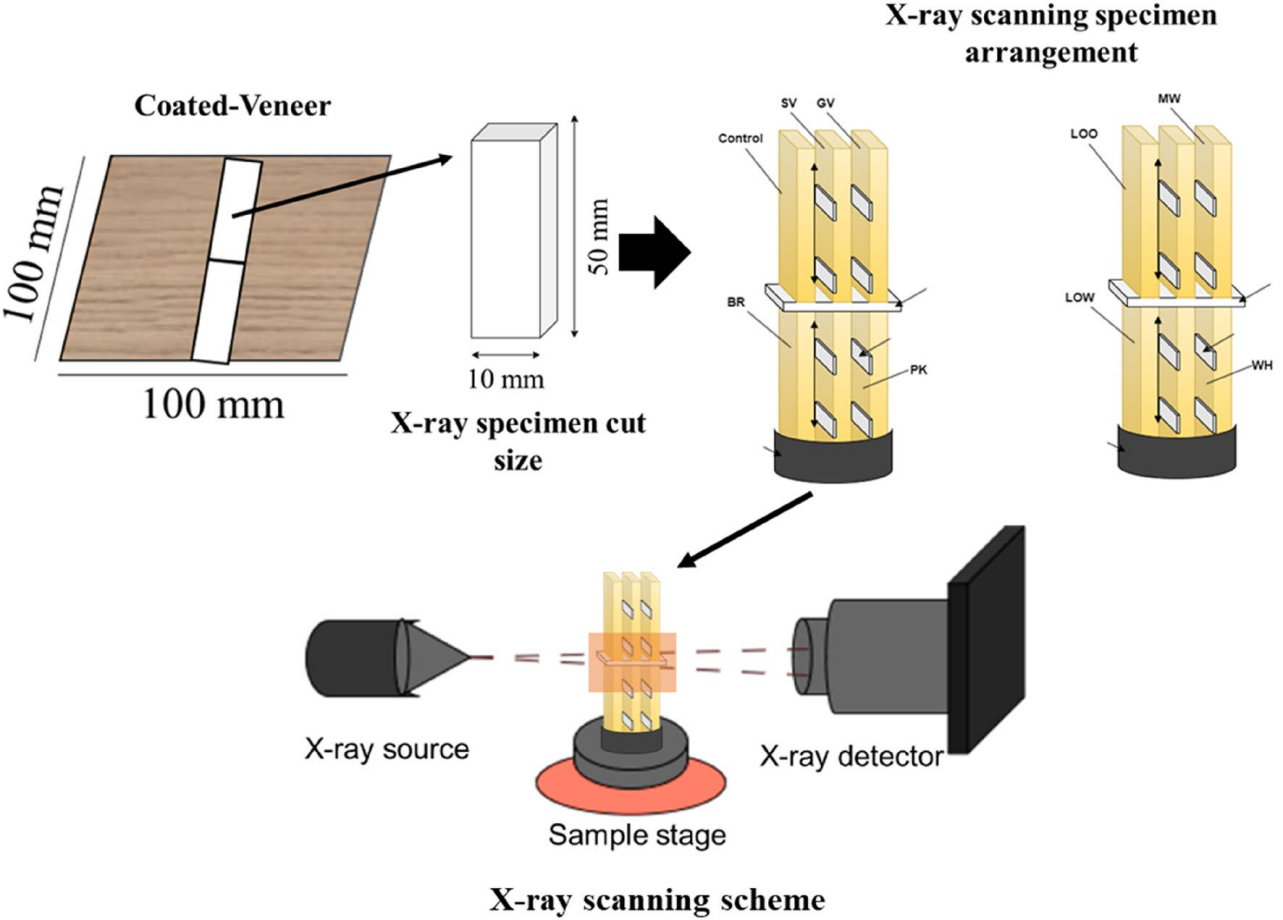
Schematic of the X-ray microtomography specimens’ preparation and scanning. The specimen arrangement consists of control, silver (SL), gold (GL), brown (BR), pink (PK), light-oak oil-based (LOO), matte white (MW), light-oak water-based (LOW), and white (WH). The two-way arrow on the scanning specimen arrangement indicating the face to face coating side and the long white bar indicating the tape. The orange square in the X-ray scanning scheme indicating the field of view of the X-ray.

The coated veneers were cut into dimensions of 10 mm × 50 mm using a table saw. Samples were selected from the middle of the veneer to avoid possible interference with the cross-section of the sample used for X-ray image analysis. The size of specimens was based on the FOV (field of view) of the apparatus which used lens L1080, FOV φ3.6 (diameter) × 2.8 (height) mm. X-ray specimens of all coating materials were scanned using an X-ray microtomography apparatus (Rigaku Nano 3DX Fuji, Japan). A Mo X-ray target with a tube voltage of 50 kV and tube current of 24 mA with a 0.1 mm thick aluminum (Al) filter (for generating X-rays with higher energy) was used. Samples were placed in the sample stage of the apparatus at a distance from the center of the sample stage to the 8 mm lens, and the stage height was adjusted as the six veneer samples; all in the field of view. The acquisition settings were binning 2 resulting in 2.2 μm/pixel, 6 s exposure time, and angular step at 0.2° during the sample rotation through 180°. The binning level used was recommended by Oishi and Tanaka^13^ to achieve a good resolution within an effective scanning duration. After scanning, the CT reconstruction process was implemented using the RX 3D reconstruction software inside the computer connected to the X-ray apparatus. A schematic of the X-ray microtomography sample, cut and scanned, is shown in Fig. 1.

### X-ray image visualization and coating penetration analysis

**Figure 2:**
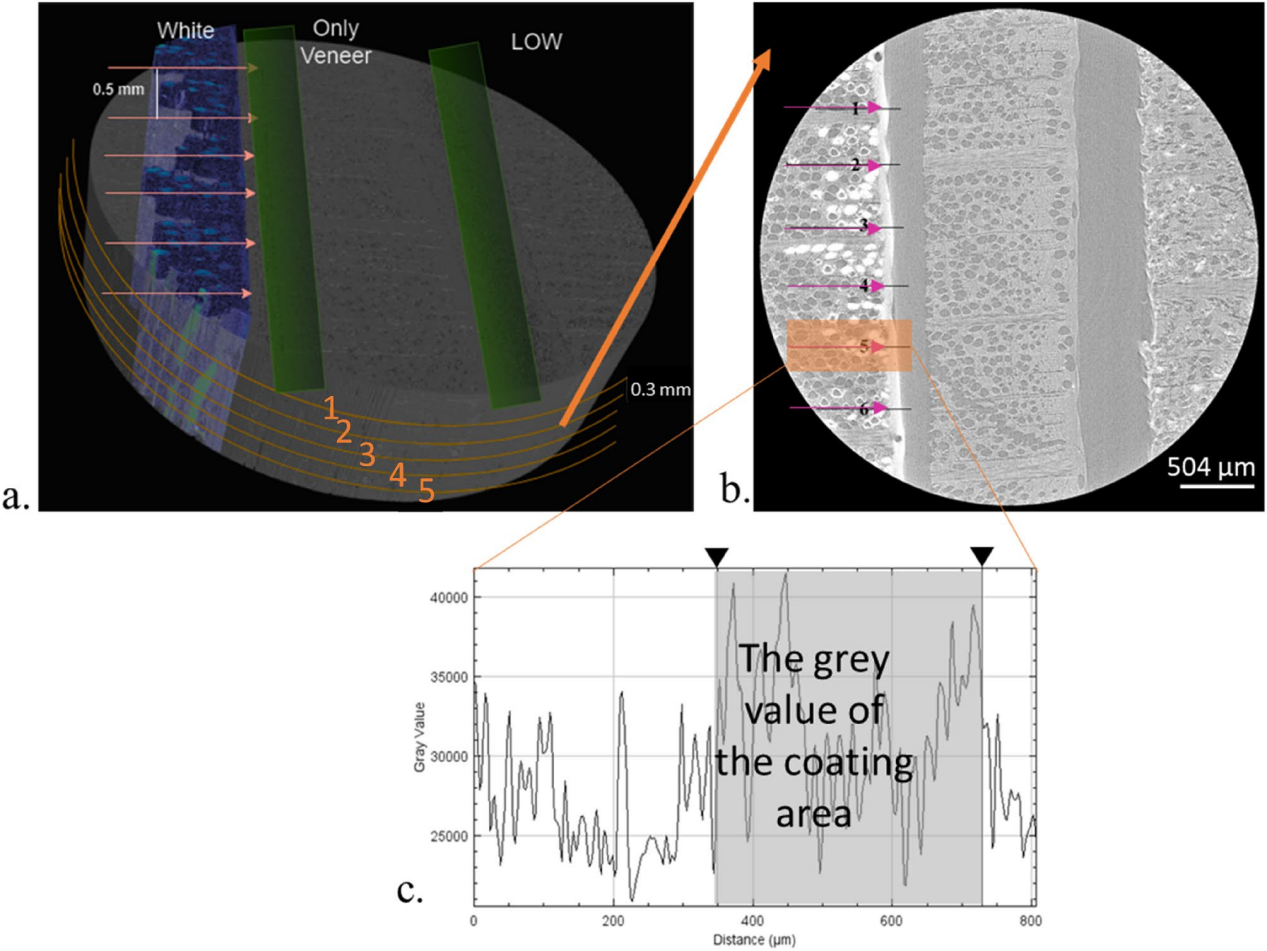
The mechanisms of coating penetration distance quantification (**a**). The three-dimensional X-ray scanning image result; determination of axis; pink arrow indicates the longitudinal axis (6 lines) and orange line indicates the tangential axis (5 lines; orange number 1–5) of coated wood; the green bar indicating the air between the specimens (**b**). Two-dimensional image from one of the tangential axis in transverse section; the pink arrow indicates longitudinal axis; orange square indicating the line that analyze using ImageJ (**c**). The grey value plot profile of the orange square.

The X-ray image stack obtained after the reconstruction process contained 1234 images with a resolution of 1648 × 1648 pixels. The three-dimensional image stack consisted of several coating materials according to the arrangement of the X-ray specimen. Coating penetration analysis was performed using the ImageJ software while processing the obtained image stack. The determination of each axis for a three-dimensional penetration plot was carried out using VGStudio Max software with the 3D image obtained, as shown in Fig. 2a. After tangential image slicing, five 2D images were obtained and numbered from one to five to perform a 2D visualization analysis to observe the appearance and disappearance of the coating tangentially. For the coating penetration analysis, the gray value on the longitudinal axis was analyzed using ImageJ to obtain the penetration depth, as explained in Fig. 2b and c.

## Results and discussion

### 2D X-ray image visualization analysis

**Figure 3:**
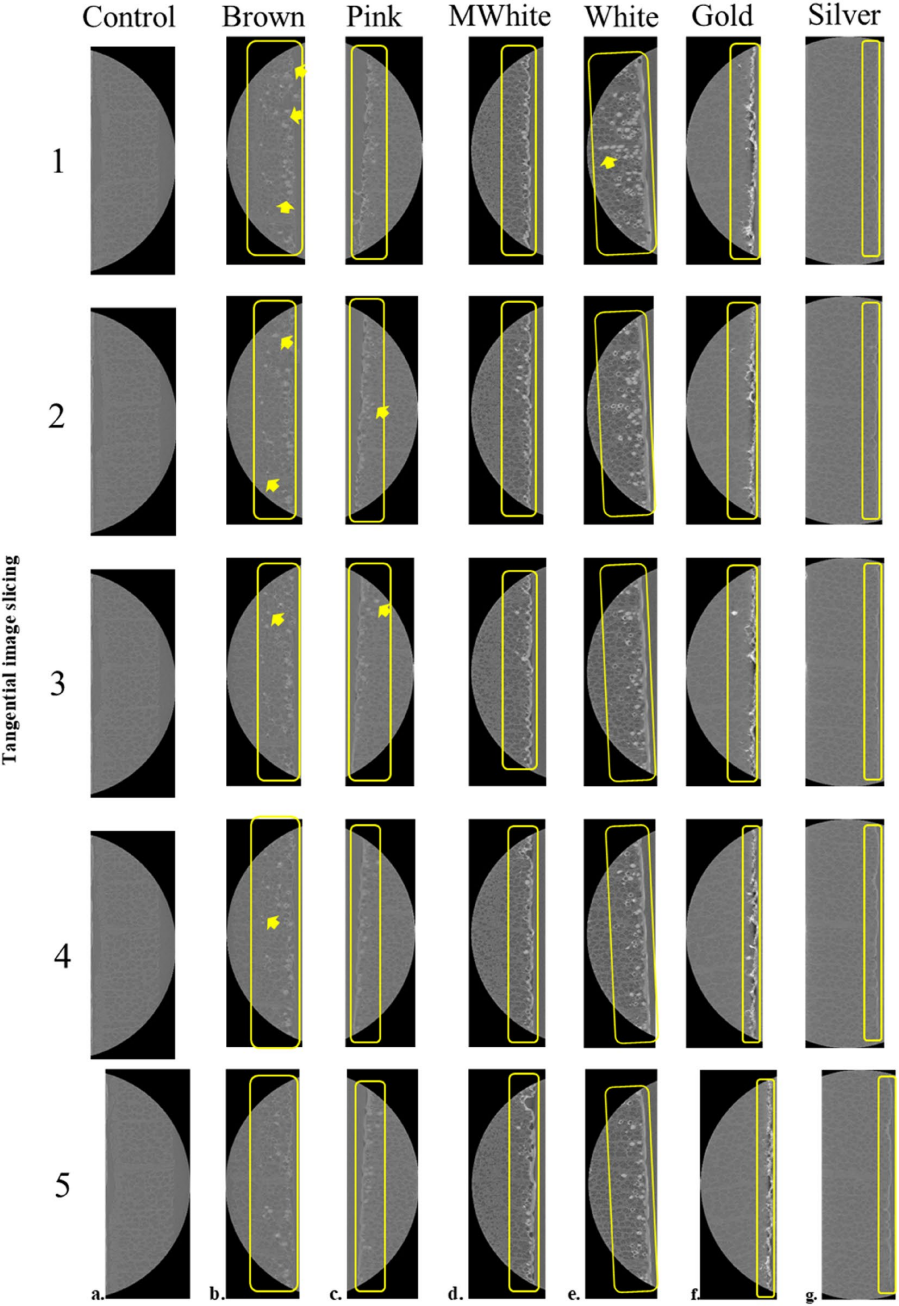
The 2D X-ray visualization of various coatings in transverse section from tangential image slicing. (**a**) Control (no treatment), (**b**) brown coating, (**c**) pink coating, (**d**) matte white coating, (**e**) white coating, (**f**) gold coating, (**g**) silver coating; yellow arrow indicating the coating existence in the wood vessels, yellow line indicating the coating area on the surface.

2D cross-sectional images (tangentially sliced) of the control and coated specimens are shown in Figure 3. The distributions of the coatings on and within the veneer were observed using X-ray microtomography. The visibility of the coating, which was successfully visualized using X-ray microtomography, could have been caused by the high number of ions in its chemical composition, which is in accordance with the results of a preliminary study by Ekaputri et al.^8^. The coating and woody materials are visible and clearly distinguished. The coating that is visible on the surface of the wood implies a white line, whereas the coating that is visible inside the wood vessels implies white dots annotated inside the yellow squares and yellow arrows in Fig. 3. This is consistent with the untreated wood image results, which do not have white lines or dots. Each coating exhibited a different phenomenon. Some coatings showed the existence of the coating both on the surface and in the wood vessels, such as brown, pink, and white coatings in Fig. 3b, c, and e, whereas the matte white, gold, and silver coatings only appeared on the wood surface (Fig. 3d, f, and g). These findings indicate that the elemental composition of the coating materials has a significant effect on the visualization. Most biological samples are largely composed of light elements such as hydrogen, oxygen, carbon, and nitrogen, which have low X-ray absorption^14^. Therefore, a coating substance with a heavier element leads to a clear visualization. In particular, the presence of elements such as Al, Fe, Cu, Zn, Si, and Ti improved the contrast between the coatings and wood cells. This image did not have any artifacts and resulted in a good image quality that was acceptable for the next step, which was quantitative analysis.

### Preliminary three-dimensional penetration plot

**Figure 4:**
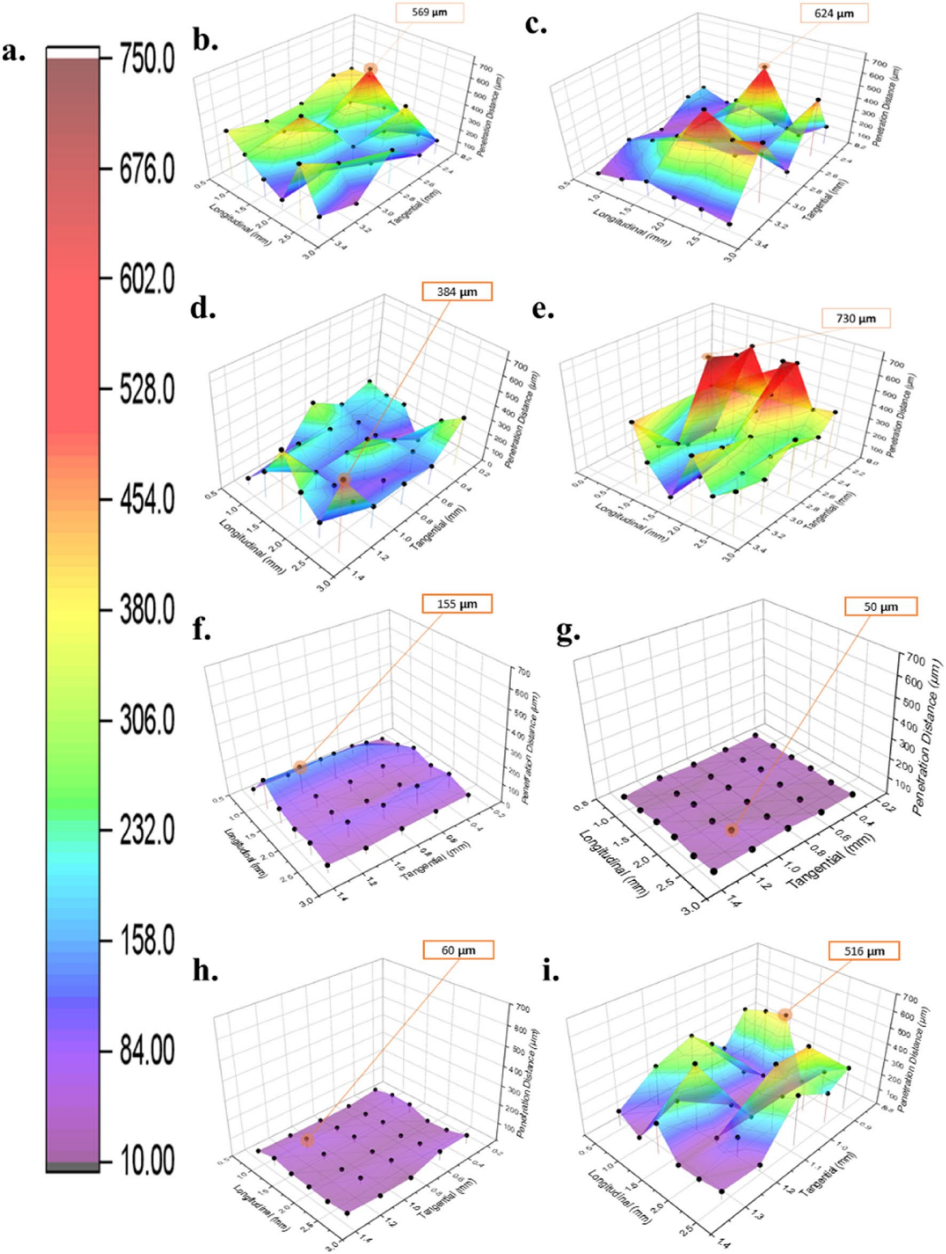
Three-dimensional coating penetration plot of Japanese commercial coating. (**a**) Color chart level of penetration depth (µm), (**b**) brown coating, (**c**) pink coating, (**d**) matte white coating, (**e**) white coating, (**f**) gold coating, (**g**) silver coating, (**h**) light-oak oil-based coating, (**i**) light-oak water-based coating.

**Figure 5:**
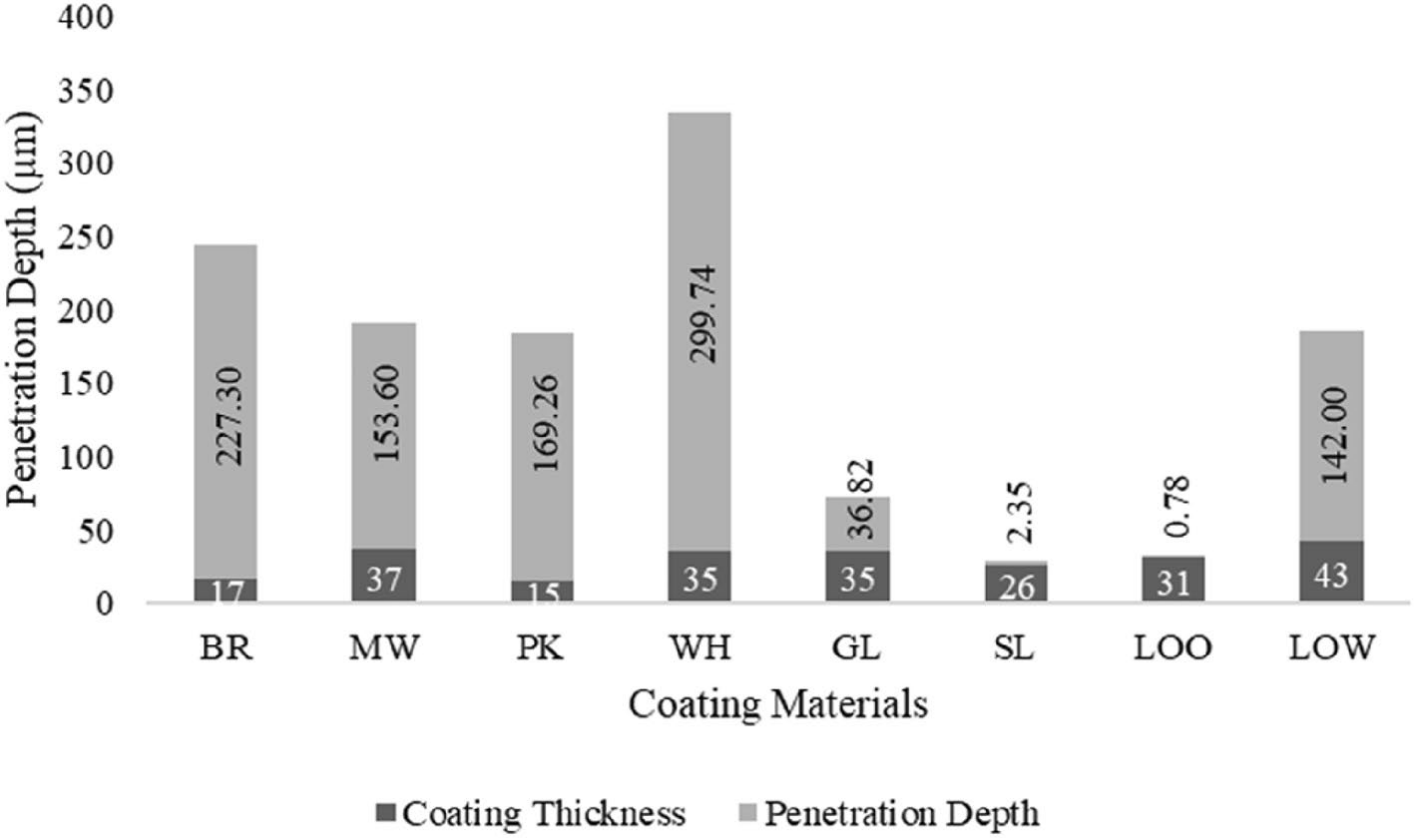
Average coating thickness and penetration dept.

A three-dimensional penetration plot is a convenient way to track the coating on and inside wood, especially in vessel sections. The preliminary analysis can quantify the amount of coating penetration at some tangential and longitudinal points (axes) by slicing the image tangentially from the 3D image stacks and calculating the penetration distance from the image and its grey value to form a 3D visual penetration plot. Figure 4 shows 3D penetration plots obtained for each coating material. The color level from purple to red indicates the different penetration depth values in µm, respectively. In addition, the average amount of coating penetration for each coating was calculated, and explained in Figure 5. The average coating penetration calculations yielded different values for each coating. The dark grey bar shows the coating thickness on top of the wood surface, whereas the light grey bar shows the coating penetration inside the wood specimens.

Regarding the coating thickness and penetration, as shown in Fig. 4, deep penetration is shown in Fig. 4b–e for the spray application method and 4i for the brush application method. Moreover, the coating that appeared almost only on the surface of the wood for both application methods is shown in Fig. 4f–h.

The coating consists of a solvent and dissolved substances. The solvent, commonly referred to as the resin, plays a crucial role in ensuring an even coating distribution by evaporating and allowing the active coating material to adhere to and penetrate the wood surface. In the case of the brush application method, it was observed that light-oak oil-based (LOO) and light-oak water-based (LOW) coating showed different phenomena. The LOO coating mainly appeared on the wood surface, whereas the LOW coating exhibited deep penetration. This phenomenon may have occurred because of the dissolved substances in the coating and the viscosity of the coating material itself. The LOO coating contains a long oil-alkyd resin and some benzene polymers, whereas the LOW coating contains a water-based acrylic resin and water, which probably affects the drying and flow rate of the coating.

Figure 4 implied that the coating containing TiO_2_ (BR, PK, and WH) was distributed evenly to a certain degree throughout the veneer, as shown by the fluctuation of the peaks. High peaks indicating the coating were distributed into the wood vessels. The coating that contained TiO_2_ with additional chemicals (MW) other than TiO_2_ (LOW, LOO, GL, and SL) was mainly present on the surface. The brown, pink, and white coatings (Fig. 4b, c, and e, respectively) had the same active chemical content, that is TiO_2_, but exhibited different penetration phenomena. The deepest penetration amount of brown, pink, and white coating was 569 µm, 625 µm, and 730 µm and this caused the white coating to have the deepest penetration compared to the brown and pink coating. Moreover, the average penetration among these coatings (Fig. 5), showed 227 µm for brown coating, 169 µm for pink coating, and 299 µm for white coating. This phenomenon could be caused by the concentration of chemical constituents in the coating materials, which affects the anchoring and penetration power of the coating itself. With the same active chemical content and the addition of silica, the matte white coating had a shallower penetration amount at 384 µm. The average penetration amount of matte white coating was 153 µm. This result implies that silica has some effect on the penetration distance, suggesting that further investigation is required to observe the interactions between silica and wood chemicals that influence the penetration rate.

In addition to TiO_2_, some coatings contain other chemicals, such as copper and zinc (gold coatings) and aluminum (silver coatings). Although the coating base was the same as the other spray coating bases used in this study, these two coatings were mainly found on the surface (Fig. 4f and g). The deepest penetration amount of these coatings was 140 µm and 50 µm. Average penetration amount for these coatings was 36 µm and 2 µm. This phenomenon likely occurred because the chemicals used in both it was difficult for the coatings to get into the wooden vessel. This was probably caused by the shape or size of the particles or the flow rate of the coating. Other constituent materials possibly influenced the drying rate of the coating, including the transfer of vapor materials (evaporation). This implies that Cu, Zn, and Al may influence the coating penetration distance, suggesting that further investigation is required.

The LOO and LOW coatings have ferric as their main active chemical with the addition of amorphous silica and cobalt for the LOO coating. On the contrary, the LOW coating has amorphous silica and TiO_2_ as additional active chemicals. Although both claimed to have the same color (light-oak), the active chemical content and resin used were different. This causes a significant difference in the penetration values as shown in Fig. 4i and h. The LOO coating had shallower penetration with 60 µm as its deepest penetration value whereas LOW had 516 µm. The average penetration amount for these coatings was 0.78 µm and 142 µm. This phenomenon implies that cobalt may affect the penetration distance, which requires further investigation.

Based on these results, various phenomenon and three-dimensional penetration plot were successfully visualized and calculated using X-ray microtomography. The active coating material significantly affected the coating penetration distance. Further investigation is required to further understand the interaction of silica and cobalt with wood materials.

**Figure 6:**
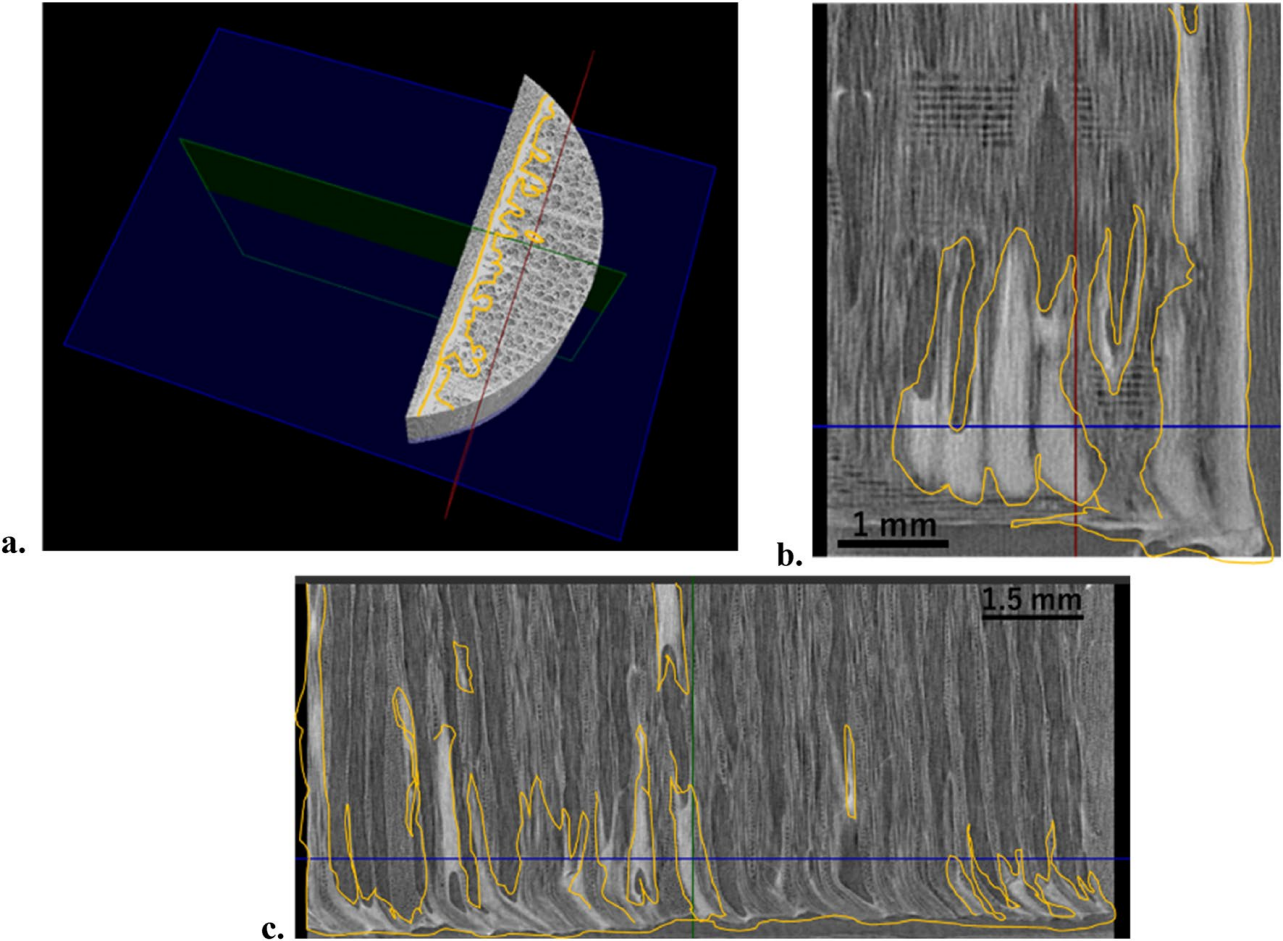
3D X-ray observation of coating penetration. (**a**) 3D image stack of veneer with white coating, (**b**) 2D representative image of a radial section were obtained from the green cut section of the 3D image, (**c**) 2D representative image of a tangential section were obtained from the red cut section of the 3D image. The yellow line annotated the coating part.

The coating penetration depth seemed to depend on the nature of the substrate and its anatomy, as shown in Fig. 3. All X-ray microtomography images were presented on the same scale. The appearance and disappearance of the coating materials inside certain vessels were successfully visualized. This phenomenon implied that the coating flowed through the wood vessels. In addition, the coating in the vessel could be verified based on the intersection of the transverse, radial, and tangential sections, as shown in Fig. 6. The blue, green, and red layers indicate the active 2D images of the transverse, radial, and tangential sections, respectively. This discovery could be used to clarify the penetration phenomena and coating quantitative analysis because the wood and coating parts could be clearly distinguished. The white parts inside the yellow lines in Fig. 6a–c indicated the coating and wood, respectively.

## Conclusions

X-ray microtomography can be used to visualize the coating penetration phenomenon in *Fagus crenata* veneers and quantify its penetration value by clearly distinguishing between the wood vessels and coating part in three dimensions. The penetration of the coating into wood vessels was successfully quantified. The three-dimensional penetration depth plot shows the unique phenomena of each coating based on its chemical content and concentration, with the highest being the white coating at 624 µm. The active chemicals in the coating materials have been implied to affect their ability to flow into wood vessels, which requires further investigation. The penetration plots quantitatively described the coating thickness and penetration depth of the coating materials to assess the quality related to the uniformity and heterogeneity of the distribution. The results described here were obtained by a non-destructive visualization technique using X-ray microtomography, which is a promising technique for observing coating penetration into woody materials for quality assurance in industrial applications.

## Data Availability

The datasets used and/or analyzed in the current study are available from the corresponding author upon reasonable request.
